# Adélie Penguin Foraging Location Predicted by Tidal Regime Switching

**DOI:** 10.1371/journal.pone.0055163

**Published:** 2013-01-30

**Authors:** Matthew J. Oliver, Andrew Irwin, Mark A. Moline, William Fraser, Donna Patterson, Oscar Schofield, Josh Kohut

**Affiliations:** 1 School of Marine Science and Policy, University of Delaware, Lewes, Delaware, United States of America; 2 Department of Mathematics and Computer Science, Mount Allison University, Sackville, New Brunswick, Canada; 3 Polar Oceans Research Group, Sheridan, Montana, United States of America; 4 Institute of Marine and Coastal Sciences, Rutgers University, New Brunswick, New Jersey, United States of America; Hawaii Pacific University, United States of America

## Abstract

Penguin foraging and breeding success depend on broad-scale environmental and local-scale hydrographic features of their habitat. We investigated the effect of local tidal currents on a population of Adélie penguins on Humble Is., Antarctica. We used satellite-tagged penguins, an autonomous underwater vehicle, and historical tidal records to model of penguin foraging locations over ten seasons. The bearing of tidal currents did not oscillate daily, but rather between diurnal and semidiurnal tidal regimes. Adélie penguins foraging locations changed in response to tidal regime switching, and not to daily tidal patterns. The hydrography and foraging patterns of Adélie penguins during these switching tidal regimes suggest that they are responding to changing prey availability, as they are concentrated and dispersed in nearby Palmer Deep by variable tidal forcing on weekly timescales, providing a link between local currents and the ecology of this predator.

## Introduction

The region surrounding Anvers Island, West Antarctic Peninsula (WAP) is a “hot-spot” for Adélie penguin activity. Adélie penguins (*Pygoscelis adeliae*) have been present in the Anvers Island region on millennial timescales [1], [2]. The presence of a pronounced submarine canyon (Palmer Deep) near this area provides a conduit for warm Upper Circumpolar Deep Water (UCDW), locally increasing primary production, which supports a productive regional food web [3]–[5]. In addition, this region has warmed significantly [6], [7] and has lost a significant amount of sea-ice [8], [9]. The Adélie penguin population in this region has decreased dramatically since the 1970’s [10], as climate conditions that support their chick rearing habitat have moved southward [11]. Understanding the interaction between the foraging behavior of the remaining Adélie penguins and physical dynamics in this historical “hot-spot” may provide insights into the future of this historic colony that has survived past warming and cooling events [12].

The effect of the tides on currents is most dramatic in coastal systems. Tidal forces interact with local geographic and bathymetric features that change sea level, cause water mass mixing, and create tidal fronts [13]–[16]. These features affect phytoplankton distribution [17]–[20], zooplankton aggregation [21]–[26], benthic grazers [27], fish behavior [28]–[30] and even marine mammal foraging activity [31]–[33]. Tidal fronts also influence seabird foraging timing and behavior by concentrating prey or providing favorable currents that regulate foraging trips. For example, short-tailed shearwaters (*Puffinus tenuirostris*) broaden their access to smaller euphausiids by foraging near recurrent tidal fronts in the Akutan Pass [34], while auklets coordinate their feeding behavior with peak tidal current velocities in the shallow passes in the Aleutian Islands [35]. In Vancouver Island, Canada, planktivorous diving birds prefer deep water with moderate to high tidal flow while benthic invertebrate feeders preferred shallow, tidally slack waters. Piscivorous diving birds feed in shallower water during moderate tidal flows and in a variety of water depths during slack water [36]. The impact of flood and ebb tidal forces has also influences the mode of transportation of Magellanic penguins (*Spheniscus magellanicus*), which avoid swimming against strong tidal currents by diving deeper or walking in San Julian Bay, Argentina [37]. Magellanic penguins also take advantage of tidal oscillations in the Beagle Channel, Argentina, to transport them to foraging locations maximize their foraging success [38]. The wide and varied exploitation of different tidal forces by sea birds show that tides produce regular and predictable concentrations of resources in an otherwise patchy coastal environment [39]. These local tidal concentrating mechanisms may become more ecologically important, as tides are not significantly affected by a changing climate.

In this study we test the hypothesis that tides are a significant predictor of Adélie penguin foraging locations in the Anvers Island region of the West Antarctic Peninsula (WAP). To do this, we used a combination of satellite-tagged Adélie penguins, historical tide records and currents derived from a Slocum glider autonomous underwater vehicle (AUV). We found a significant relationship between tidal regime and Adélie penguin foraging location during our field season and used an additional nine years of penguin location data to test the historical robustness of our results.

## Methods

### Penguin ARGOS Tags and Dive Recorders

**Figure 1:**
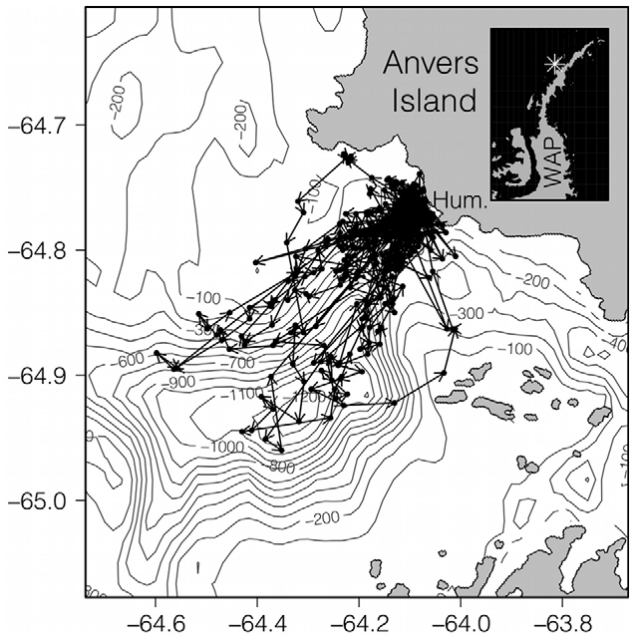
Filtered satellite tracks from Adélie penguins located at Humble Is. (Hum.), on the South coast of Anvers Island (white asterisk on inset) on the West Antarctic Peninsula (WAP) in January 2011. These birds also carried dive recorders. Arrows represent the path of foraging trips. Contours are bottom bathymetry (m) showing the location of Palmer Deep.

From January 5–27, 2011, we tagged 11 Adélie penguins at the Humble Is. rookery near Palmer Station, Anvers Is., Antarctica (64° 46′ S, 64° 04′ W). This study area is characterized by large changes in bathymetry near shore, and narrow fjords characteristic of the WAP (Figure 1). Penguins selected for tagging were paired and had brood-stage nests containing two chicks. We use the brood stage as a “biological standard” to control for changes in parental foraging behavior that might be affected by chick age [40]. Tags were a custom mold based on SPOT and SPLASH tag configurations from Wildlife Computers (Redmond, WA, USA). Our tags had a sloped frontal area of 17×18 mm (306 mm^2^), weighed 55 g and had an antenna length of 12 cm. Tag length was 86 mm. All tags in the 2011 season were equipped with pressure sensors to measure penguin dive depths (TDR, Lotek Wireless). Dive data was recorded at 1 Hz. Tags were fastened to anterior body feathers using double sided tape and small plastic cable ties. Tags were rotated to new penguins every 3–5 days depending on fair weather conditions allowing for access to the colony. The tag represents less than 2% of body mass of the lightest penguins that are typically tagged (range 3.2–4.7 kg). Some devices can affect foraging trip duration [41], [42]; our study is focused on foraging location rather than trip duration. Furthermore, tags that have been shown to affect penguin foraging trip duration were in some cases up to three times heavier and had double the frontal area when compared with our custom tags [41], [42]. Our tags are also among the lightest available and typically deployed for only 3–5 days before removal and rotation to other birds. We did not test explicitly for a “tag effect” on our penguins, but considering the size of the tag, we expect any effect to be small. Location-only data were collected from 103 Adélie penguins for ten breeding seasons (Dec–Feb) between 2002 and 2011 (Table 1) using similar tags and procedures.

**Table 1:** The deployment dates, number of ARGOS locations and mean range of the Adélie penguins tagged in each season.

Season	No. Birds	Dates Deployed	No. ARGOS locations (Post-filtering)	Mean range km (± s.d.)
2002	4	2002-01-19–2002-02-07	300 (256)	24.99 (±16.17)
2003	23	2002-12-28–2003 -02-11	2550 (1811)	11.29 (±7.56)
2004	22	2004-01-04–2004-02-08	2627 (1790)	7.72 (±5.12)
2005	19	2005-01-06–2005-02-07	1352 (991)	9.90 (±6.36)
2006	3	2006-01-18–2006-01-21	148 (119)	26.16 (±29.08)
2007	8	2007-01-11–2007-02-05	481 (340)	8.74 (±5.10)
2008	5	2008-01-19–2008-02-06	468 (367)	7.99 (±5.04)
2009	5	2009-01-06–2009-01-21	482 (445)	8.61 (±4.90)
2010	11	2010-01-12–2010-02-01	543 (478)	7.62 (±4.62)
2011	13	2011-01-05–2011-01-27	765 (690)	8.76 (±7.21)
2011*	11	2011-01-05–2011-01-27	729 (668)	11.31 (±7.86)

* Birds that recorded dive information in addition to ARGOS location.

### Penguin Location Data Filtering

The quality of the location data depends on how many ARGOS satellites are in view while the tag is above the water. The porpoising and diving behavior of traveling penguins can result in poor quality location data. Location data qualities are classified as 3, 2, 1, 0, A, and B under the least-squares ARGOS algorithm. Class 3, 2, and 1 positions are accurate within 100 m, 250 m, 500 m−1500 m respectively. Class 0, A, and B positions are locations that have no error estimation [43]. We controlled the quality of our location data using three steps. First, we eliminated erroneous terrestrial positions using land masks from the National Snow and Ice Data Center, Atlas of the Cryosphere (http://nsidc.org/data/atlas/news/antarctic_coastlines.html). Second, we applied a sequential filter that considers location data quality flags and distance between successive locations based on maximum sustained swimming speed of the penguins [44] using the R argosfilter package [45]. Our threshold swimming speed was based on a maximum sustained swimming speed of 8 km hr^−1^ [40], [46]. Finally, we visually inspected each track and manually removed any class B points that were unreasonable based on coastal geometry. For example, the distance filter considers only great circle distances and does not take into account geographic barriers such as islands, which would increase the travel time between points (Table 1).

### Dive Records

Dive records from 2011 were zero-offset using the diveMove package in R [47]. Based on previous studies of penguin diving behavior, we considered dives deeper than 5 m to be foraging dives [48], [49]. diveMove uses recursive filtering and a diving threshold to correct for drift in TDR depth sensors and identify diving behavior. This approach has been used to correct diving records of King penguins (*Aptenodytes patagonicus*) [50]. The dive records and penguin location data were then time merged. Location data within 150 seconds of a dive identified by the diveMove software were identified as foraging locations. Assuming a maximum swimming speed of 8 km hr^−1^ [40], [46], diving must have occurred within one third of a kilometer of a location fix.

### Tidal Measurements and Classification

A tide gauge mounted on the pier at Palmer Station, Anvers Island, Antarctica, recorded tidal amplitude during our experiment. The tide gauge is 1.7 km from Humble Is. We classified the tidal forcing regimes as diurnal or semidiurnal based on counting the number of high tides in a day. Time periods with one high tide per day were classified as a diurnal regime and all other tidal time periods were classified as a semidiurnal regime.

### Depth Integrated Currents from a Slocum Glider

We deployed a Slocum electric glider AUV in two successive missions from January 10–14, 2011 for a 62 km mission and January 15–31, 2011 for a 178 km mission. These vehicles have previously been used to provide environmental context for penguin foraging behavior [51]. Gliders are buoyancy driven and travel in an underwater “saw-tooth” pattern [52] between 1 m and 100 m, with surface GPS fix every 2 hours or upon reaching a waypoint. While underwater, the glider used internal compass heading corrected for declination to navigate to its next waypoint. Integrated currents between glider surfacings were estimated by the difference between where the glider surfaced based on a GPS fix, and the estimated location of the glider based on internal navigation. This method produced a 100 m depth integrated current estimate every two hours of the glider mission. During this experiment, the glider estimated currents in the general area of the Palmer Deep, which is a historically important location for penguin activity [3]. Twice, during a diurnal and a semidiurnal tidal regime, the glider was programed to remain near a station (“station keep”) at the northeast edge of the Palmer Deep to resolve the temporal changes in currents over a diurnal and semidiurnal tide cycle.

### Analysis of Penguin Foraging Location

We tested the hypothesis that Adélie penguins forage at different locations different tidal regimes (diurnal vs. semidiurnal) by using a linear mixed model [53] on locations merged with dive information in the 2011 season. Because successive locations and dives for each penguin are spatially auto-correlated, we divided the location records associated with diving behavior into trips. Trips were defined as a set of locations separated by return (within 0.5 km) to Humble Is. We then treated each trip as a random effect and tidal regime as a fixed effect in a linear mixed model. We also developed models that included tidal amplitude and Julian day as fixed effects to account for influences of short term (flood and ebb tide) and possible intra-seasonal dependencies on penguin location We repeated this analysis for location-only data (2002–2011), even though we could not distinguish diving locations from non-diving locations. We expect qualitative similarity to results from the 2011 season where diving locations can be separated from non-diving locations, however quantitative differences in estimated fixed effects are expected. To visualize differences in penguin location between tidal regimes, we used a two-dimensional kernel density filter with a grid cell of 725 m. The size of the Gaussian smoothing kernel was ∼3 km.

## Results

### Penguin Locations and Dive Records

The ARGOS filtering technique removed 24% of the ARGOS locations for all years (Table 1). In 2011, we collected 738 hours of dive depth records from 11 Adélie penguins. We classified 201 locations as diving locations and 467 locations as non-diving locations. The 2011 record was separated into 30 trips, while the historic record was separated into 603 trips. The Adélie penguins from Humble Is. frequently forage over the northeast edge of Palmer Deep (Figure 1) and follow noticeably different trajectories during the diurnal and semidiurnal tidal regime are evident (Figure S1).

### Palmer Tide Records

**Figure 2:**
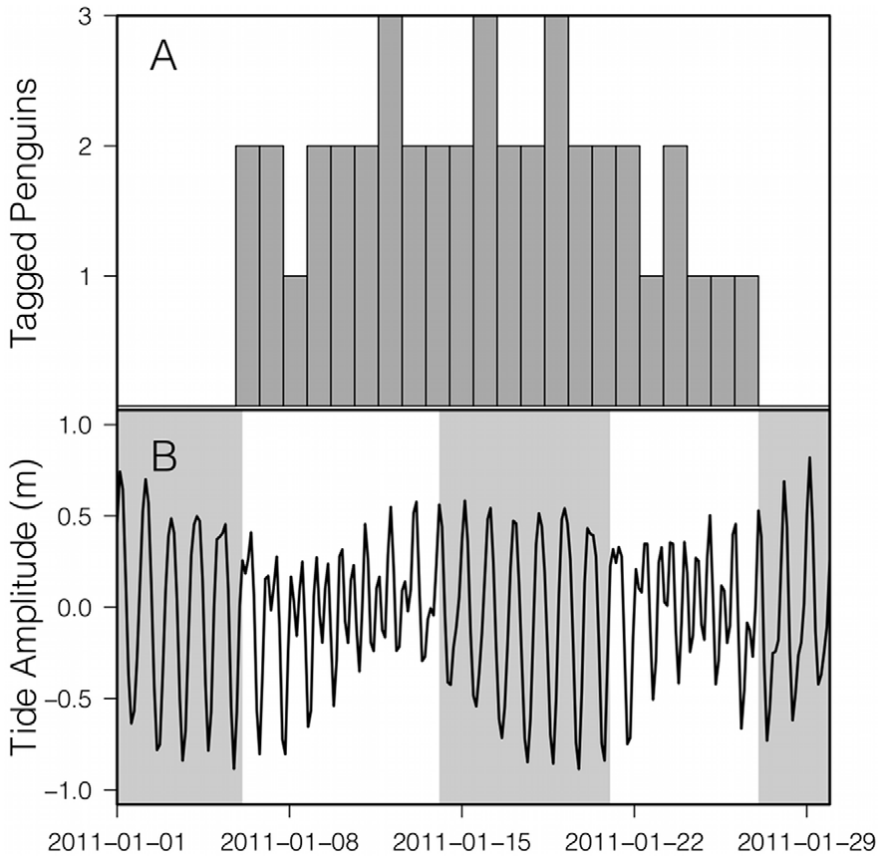
The number of tagged Adélie penguins deployed each day over the course of the 2011 experiment compared to the tidal record at Palmer Station. The number of penguins tagged was between 1 and 3 during the 2011 experiment (A). Mixed tide cycles at Palmer Station during the field season showing the shift from diurnal to semidiurnal tides. Diurnal tides are shaded in grey (B).

The tides at Palmer station are mixed, and switch between diurnal (one high and one low tide per day) and semidiurnal (two highs and two lows per day) (Figure 2). The principal tidal constituents are the diurnal K_1_ and lunar O_1_ and the semidiurnal K_2_ and M_2_ [54]. Mean tidal amplitudes during our study were 1.23 m and 0.93 m during diurnal and semidiurnal tidal regimes respectively. During the 2011 season, 32% of the penguin trips were during the diurnal tidal regime, while 68% were during the semidiurnal tidal regime. For all penguin trips from 2002–2011, 46% were during diurnal and 54% were during semidiurnal tide regimes.

### Currents in Palmer Deep

**Figure 3:**
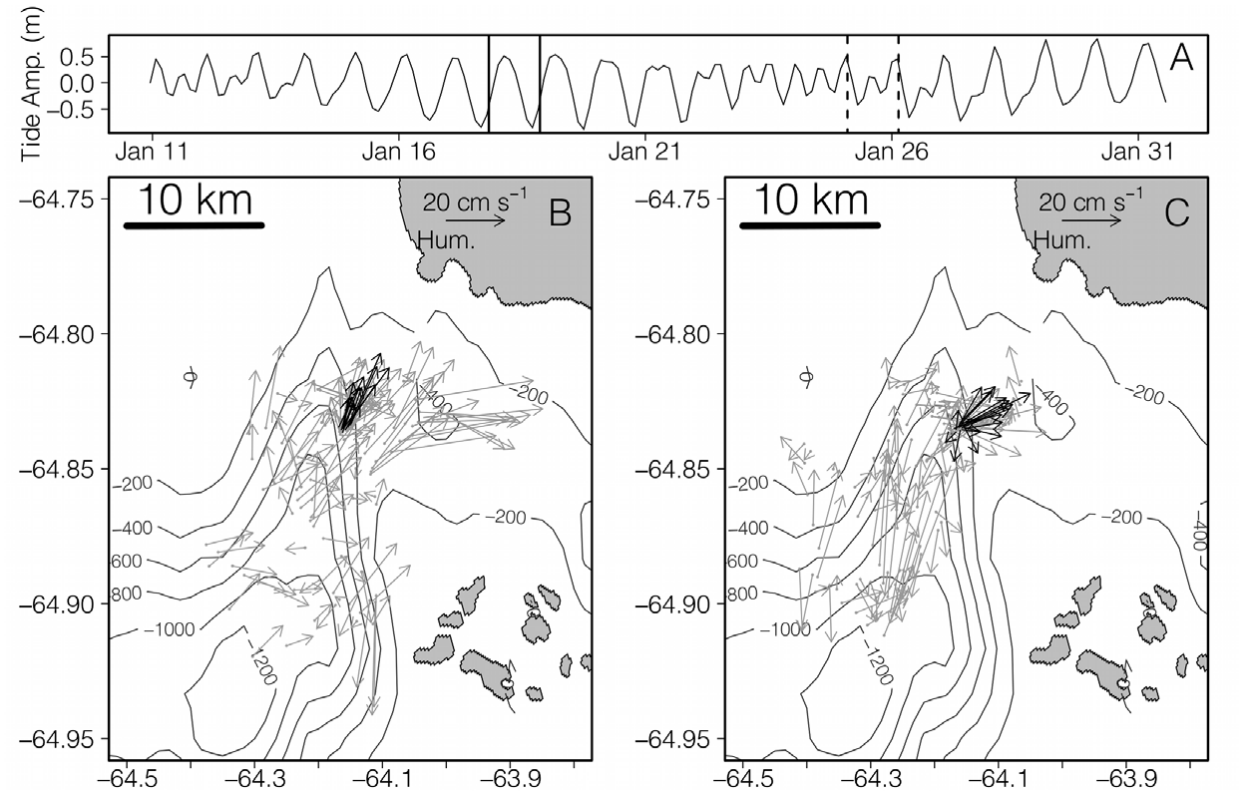
Depth integrated currents measured by a Slocum Glider AUV deployed for the month of January 2011 (arrows). Flow in both tidal regimes is complex, but onshore toward the northeast edge of Palmer Deep in both tide regimes throughout the glider mission. The two separate “station-keeping” periods during strong diurnal tides (station keeping between black lines) and semidiurnal tides (station keeping between dashed lines) are shown in panel A. Black arrows represent the currents measured while station keeping during diurnal and semidiurnal in panels B and C respectively. During the diurnal tides, flow was always toward the northeast edge of the canyon showing no reversals. During the semidiurnal tide, flow oscillated between shoreward and offshore flow, however the shoreward flow was much stronger.

**Figure 4:**
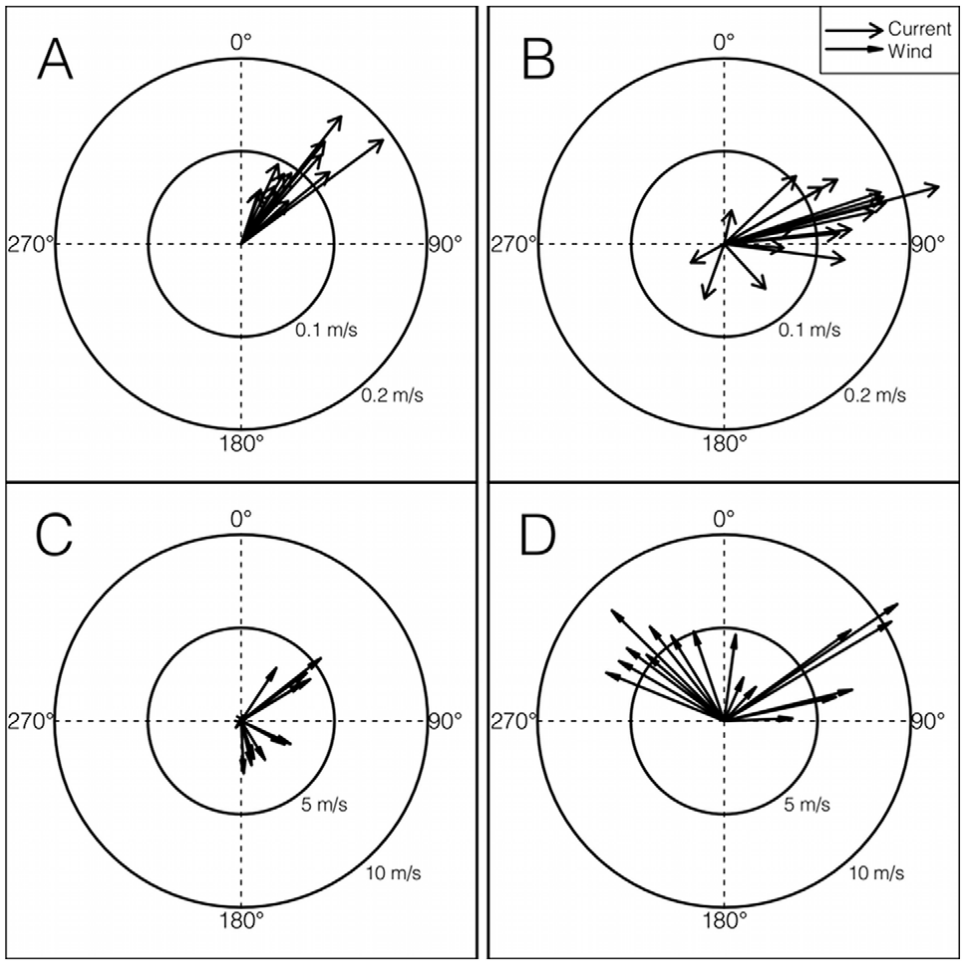
Depth integrated currents and surface winds during AUV station keeping. During diurnal (A) tidal regime, currents never reversed. Currents during a semidiurnal tidal regime reversed for part of the tidal cycle (B). Winds during the diurnal (C) and semidiurnal (D) tidal regimes were uncorrelated to current flow during while the AUV was station keeping.

In 2011, the AUV made 130 and 122 estimates of 100 m vertically integrated currents during diurnal and semidiurnal tides respectively. The mean current speed was 0.13 m s^−1^ with a range of 0–0.41 m s^−1^. Currents directed toward the northeast, southeast, southwest and northwest quadrat were 59%, 19%, 11%, and 10% of all current observations, indicating a general flow towards the northeast edge of Palmer Deep (Figure 3). Mean current velocities during diurnal and semidiurnal regimes were 0.14 (s.d. ±0.09) and 0.11 (s.d. ±0.08) m s^−1^ respectively. A t-test showed that currents during diurnal tides were significantly stronger than currents during semidiurnal tides (t = 3.10, d.f. = 247.84, p = 0.002). During diurnal tides, current bearings were toward the northeast, southeast, southwest and northwest quadrats 68%, 16%, 7%, 9% as compared to 50%, 22%, 14%, 11% during semidiurnal tides indicating stronger flow toward the northeast edge of Palmer Deep, near Humble Is. more often during diurnal tides. The distribution of current bearings between the two tidal regimes was significantly different according to a Mardia-Watson-Wheeler test (W = 13.988, p<<0.001) [55]. The non-parametric Mardia-Watson-Wheeler test was necessary to test for differences in current bearing between the tidal regimes because the current bearing distribution did not follow a von-Mises (circular normal) distribution. During one diurnal tidal regime, and one semidiurnal tidal regime, the glider maintained its position (“station-kept”) to measure currents over a tidal cycle at the northeast edge of Palmer Deep (Figure 4). During semidiurnal tides, the tidal currents are asymmetric over a tidal cycle with stronger currents directed up the canyon. However, during diurnal tides, there is no current to the southwest, indicating that the direction of flow is steady towards the northeast edge Palmer Deep and Humble Is. throughout the tidal cycle. The current bearings between the tidal regimes during the station-keeping missions were significantly different according to a Mardia-Watson-Wheeler test (W = 21.009, p<<0.001). Wind speeds at Palmer Station while station keeping were weak (mean 2.92±1.45 m s^−1^ and 6.35±2.41 m s^−1^ during the diurnal and semidiurnal tides respectively) and uncorrelated to the vertically integrated currents measured by the AUV during both the diurnal (t = 1.0, d.f. = 11, p = 0.34) and the semidiurnal (t = −0.04, d.f. = 15, p = 0.96) tidal regimes. This indicates that wind speed had little effect on the 100 m depth integrated currents during the station-keeping experiments of the AUV. The significant difference of tidal current bearing between the diurnal and semidiurnal regime provide justification for treating the two tidal regimes as factors in our statistical models.

### Analysis of 2011 Penguin Diving Locations

**Figure 5:**
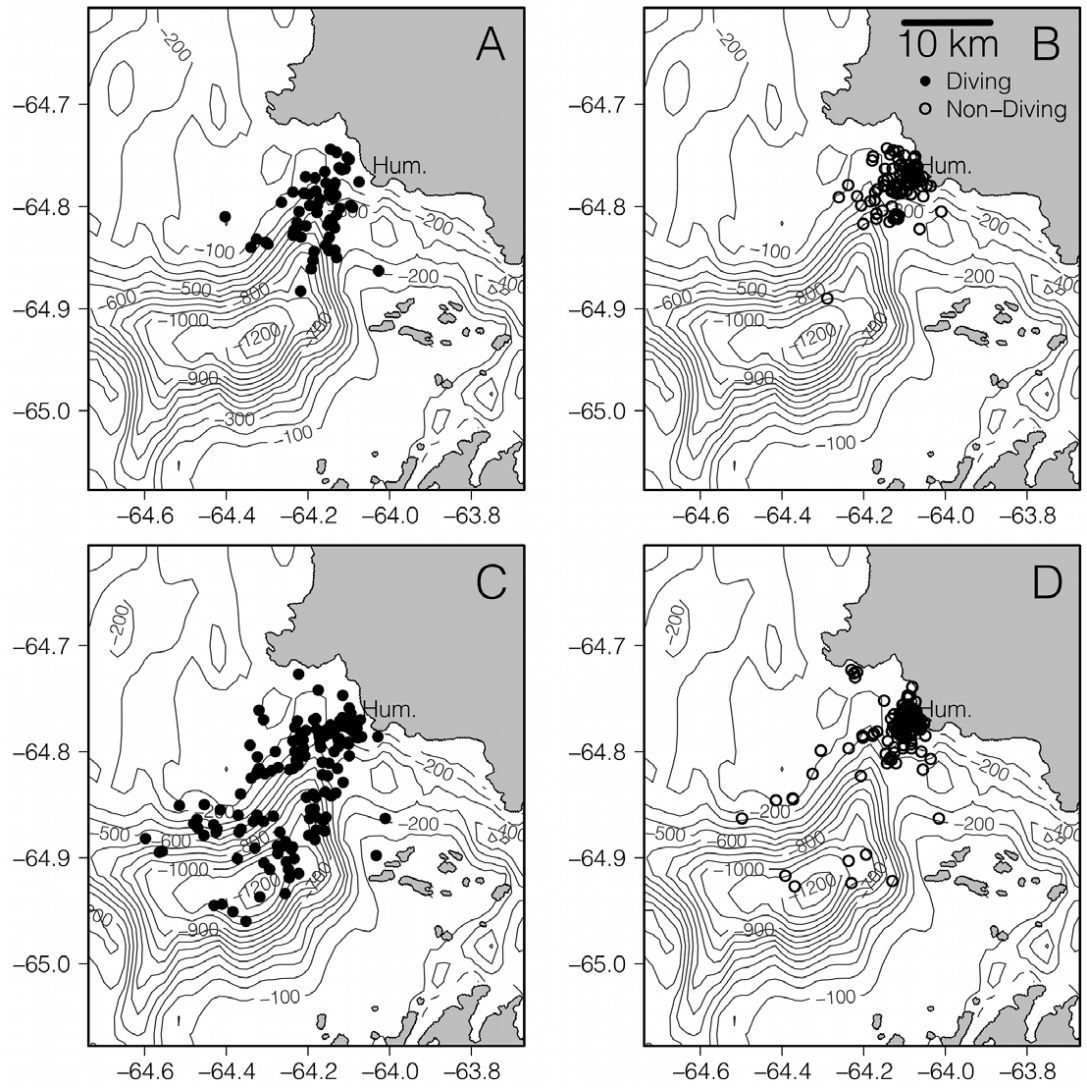
Penguin locations corresponding to diving and non-diving behavior during the 2011 season. Panels A and B are during the diurnal tidal regime and diving panels C and D are during the semidiurnal tidal regime.

We used a linear mixed effects model fit by maximum likelihood to estimate the relationship between tidal regime and the penguin locations relative to their rookery on Humble Is.:
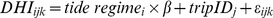
(1)where DHI is the distance of the penguin location to Humble Is., tide regime (i = 1, 2) is a fixed effect factor that corresponds to the diurnal or semidiurnal regime, β is an estimated coefficient, tripID (j = 1, 2, 3…) is a random effect and ε_ijk_ is the residual error, assumed to be normally distributed (k = 1, 2, 3…). This model showed that locations associated with diving behavior were significantly farther from Humble Is. during the semidiurnal tide regime compared to a diurnal tide regime (Figure 5) in 2011. The mean (± S.E.) distance to diving locations from Humble Is. was 5.4±1.4 km during the diurnal tidal regime while the mean distance to diving locations from Humble Is. was 9.1±1.5 km during the semidiurnal tidal regime (AIC = 1284, t = 2.50, p = 0.015). The residuals of this model satisfied the assumption of normality. Since non-diving locations are generally co-located with diving locations (Figure 5), we repeated the analysis on the 2011 data, for all diving and non-diving locations. The residuals of a linear mixed model that included all locations from 2011 indicated that the data were not normally distributed. Therefore, we log_10_ transformed DHI, after which model residuals were nearly normal. We found that penguin locations, irrespective of diving behavior, were significantly farther from Humble Is. during a semidiurnal tide compared to a diurnal tide (AIC = 345.3, t = 2.99, p = 0.004). The mean (±SE) distance to Humble Is. during a diurnal tide was 2.7±0.5 km and the mean distance to Humble Is. during a semidiurnal tide was 4.4±0.3 km. Mean distances are closer to Humble Is. when all locations are considered, compared to the diving-only locations. This is likely a consequence of not separating locations that are associated with diving behavior.

Changes in penguin foraging distance and location have been observed to vary with the season [56], [57], and with daily flood and ebb tides [38]. To test for intra-seasonal changes or changes related to flood or ebb tides in the penguin location distance from Humble Is. we also included Julian day and tidal amplitude as additional fixed effects in the linear mixed effects models:
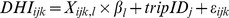
(2)where DHI is the distance of the penguin location to Humble Is., *X* is a three column (l = 1, 2, 3), fixed effects design matrix of the tide regime (i = 1, 2), tidal amplitude (m) and Julian Day (d) and β is a vector of three estimated coefficients of the fixed effects. tripID (j = 1, 2, 3…) is the random effect and ε_ijk_ is the residual error, assumed to be normally distributed (k = 1, 2, 3…).

Similar to Eq. (1), model fits of Eq. (2) showed that penguin diving locations (AIC = 1284, t = 2.68, p = 0.008) and all penguin locations in 2011 (AIC = 345, t = 2.75, p = 0.003) during the diurnal tide regime were significantly closer to Humble Is. compared with the semidiurnal tidal regime. Neither tidal amplitude nor Julian day were significant predictors of DHI for diving locations alone (tidal amplitude t = 1.19, p = 0.235; Julian Day t = −0.75, p = 0.941), nor all penguin locations (tidal amplitude t = 1.14, p = 0.254; Julian day t = −1.18, p = 0.237) in 2011. An AIC comparison of the model fits of Eq. (1) and (2) showed they were not significantly different for penguin diving locations (d.f. = 2, χ^2^ = 1.41, p = 0.495), or for all penguin locations (d.f. = 2, χ^2^ = 2.490, p = 0.288), indicating that there is not an effect of tidal amplitude or Julian day on penguin foraging location in 2011.

### Analysis of 2002–2011 Penguin Locations

**Figure 6:**
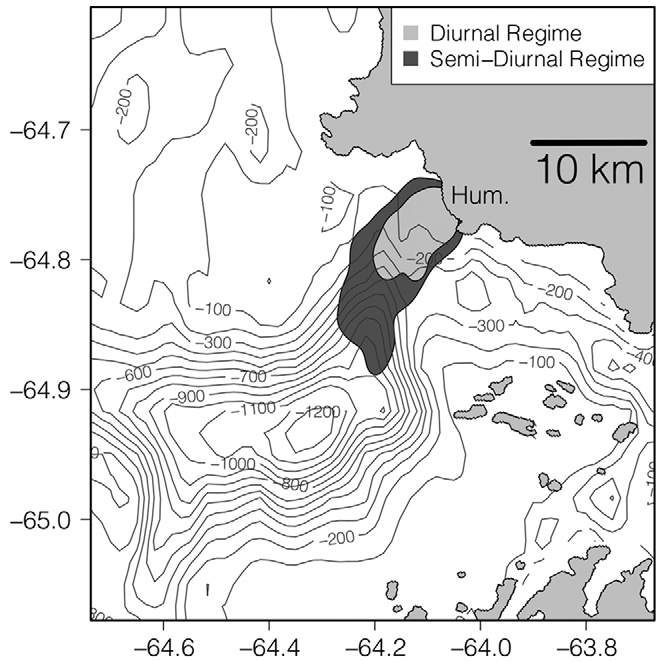
The locations of Adélie penguins during the diurnal and semidiurnal tidal regimes or ten seasons (2002–2011). Ninety five percent of Adélie penguin locations were within the light grey contour during diurnal tides and within the dark grey contour during semidiurnal tides indicating that Adélie penguins tended to be farther from Humble Is. during semidiurnal tides.

Location-only data from 2002–2011 also show a difference between penguin locations between tidal regimes. Contours containing 95% of observations showed that penguins used a smaller area to forage during diurnal tides (40.6 km^2^), compared to semidiurnal tides (101.4 km^2^) (Figure 6). A linear mixed effects model the same form as Eq. (1) on log_10_ transformed distance data showed that penguins were significantly farther from Humble Is. during semidiurnal tides, compared to diurnal tides (AIC = 13263, t = 2.054, p = 0.04). The mean distance (±SE) from Humble Is. during diurnal tides was 2.02±0.11 km and 2.34±0.08 km for semidiurnal tides. Although significant differences in penguin distance from Humble Is. are observed (Figure 6), the inability to identify diving locations in seasons prior to 2011 likely occludes the true spatial separation of penguin diving behavior across tidal regimes.

We also tested the effect of tidal amplitude and Julian day on DHI for the 2002–2011 location data using Eq. (2) as the model. Neither tidal amplitude (t = −0.524, p = 0.601) nor Julian day (t = −0.195, p = 0.845) was a significant predictor of DHI. An AIC comparison of Eq. (1) and Eq. (2) using location-only data from 2002–2011 showed that the inclusion of tidal amplitude or Julian day as a predictor of DHI did not significantly change AIC (d.f. = 2, χ^2^ = 0.303, p = 0.859), indicating that tidal regime alone was the best predictor of penguin location.

## Discussion

Our results show that weekly switching in tidal regimes, but not daily changes in tidal amplitude, is a significant predictor of Adélie penguin foraging locations near a historic penguin “hot-spot” that is characterized by deep submarine canyons and fjords. By comparison, the foraging location of Magellenic penguins that also inhabit the fjord rich environment of southern Chile, is best predicted by daily changes in tidal amplitude and current direction [38]. The contrast in response to tidal forces between these two penguin groups can only be understood in the light of the hydrography of their respective locations. An AUV consistently occupied the general foraging area of Adélie penguins, and showed that the bearing of the tidally driven flow patterns over Palmer Deep did not follow a daily oscillation, but rather oscillated between weekly tidal regimes (Figure 4). The link between both penguin diving and non-diving location and tidal regime switching is strongly supported statistically for both the 2011 field season (Figure 5), and for historical observations of Adélie penguin location (Figure 6) indicating that the weekly switching of tidal regime in our region plays a strong role in organizing the local coastal ecosystem near Palmer Deep.

The interaction of nutrient rich UCDW with switches in tidal regime has already been observed in primary producers near Palmer Deep. Phytoplankton concentrations are much higher near the Adélie penguin colonies during diurnal tides compared to semidiurnal tides [20], indicating that both the presence of the Palmer Deep and tidal regime switching impact the base of the food web. While we did not have direct krill observations during our 2011 field experiment, our observations of current magnitude and direction suggest that krill may also be differentially concentrated during different tidal regimes. During our experiment, the 100 m depth integrated flow measured by the AUV was predominantly northeast (59% of all depth integrated current measurements) toward the head of Palmer Deep suggesting water in our study area is continually being replaced by water from the continental shelf. The mean speed of depth integrated currents was 0.13 m s^−1^, which is about half of the normal swimming velocity for krill [58], the predominant prey item in the region [59]. Also, in the summer season, krill are generally located in the upper 100 m of the water column [60]. Therefore the direction of the flow regime would influence the location of krill populations over Palmer Deep. During the diurnal tidal regime the flow towards the northeast edge of Palmer Deep never reversed, while there was only weak reversal during semidiurnal tides (Figure 4). Because tides in this region may stay in a diurnal or semidiurnal regime for up to a week, the diurnal tide regime would continually concentrate krill and other prey items at the northeast edge of Palmer Deep, near Humble Is. During the semidiurnal tide regime, the currents over Palmer Deep reverse for a portion of the tidal cycle, reducing this proposed concentration mechanism near the northeast edge of the canyon. We speculate that the reason Humble Is. Adélie penguins do not travel as far from Humble Is. during the diurnal tide compared to the semidiurnal tide is because krill are concentrated near Humble Is. by currents during the diurnal tidal regime.

It is difficult to tell from our data if the differences in Humble Is. Adélie penguin foraging locations are due to a physiological or behavioral constraint on the penguins, or if the penguins are following changing prey fields over Palmer Deep. Physiological and behavioral constraints seem unlikely, since the foraging distances of these particular Adélie penguins are short in comparison to other known breeding colonies that have foraging ranges of up to 100 km [40]. Also, the difference in mean current speeds between tidal regimes is only ∼1% of the penguins’ maximum sustained swimming speed indicating the direction of tidal currents are unlikely to have a large effect on the distance Adélie penguins forage from Humble Is.

Understanding the interaction between Palmer Deep and tidal regime switching as a potential prey concentrating mechanism has significant implications for understanding the future of Adélie penguins in this region. The climate driven southward translocation of Adélie penguin chick rearing habitats on the WAP [11] could be ameliorated by predictable, hydrographically concentrated food resources that allow Adélie penguins to persist in this region despite climactic change [12]. However, because the proposed concentrating oscillates with roughly weekly switches of tidal regime, and not daily scales of tidal amplitude, successive Adélie penguin breeding seasons do not have equal proportions of diurnal and semidiurnal tides. For example, in January 2003, 60% of the tides were during the diurnal regime, while in January 2008, 40% of the tides were during the diurnal regime. Uncovering the mechanics of this effect on krill will require more detailed surveys of local currents and krill densities to determine if the seasonal heterogeneity of tidal regime is a significant factor for Adélie penguin foraging in this region. Whether or not local hydrographic processes that concentrate prey items will provide local a refuge for Adélie penguins in a changing climate is unknown. A path forward could include the interaction between foraging and local hydrography in climate models, however downscaling these models to capture local dynamics present significant challenges [61], [62].

## Supporting Information

Figure S1 **Filtered Adélie penguin satellite tracks from January 2011. Panels A and B are tracks during diurnal and semidiurnal tidal regimes.**
(TIF)
