# Supplementary figures and images for: Adélie Penguin Foraging Location Predicted by Tidal Regime Switching
Source: PLoS One. 2013 Jan 30;8(1):e55163. doi: 10.1371/journal.pone.0055163 (PMC3559330; doi:10.1371/journal.pone.0055163)

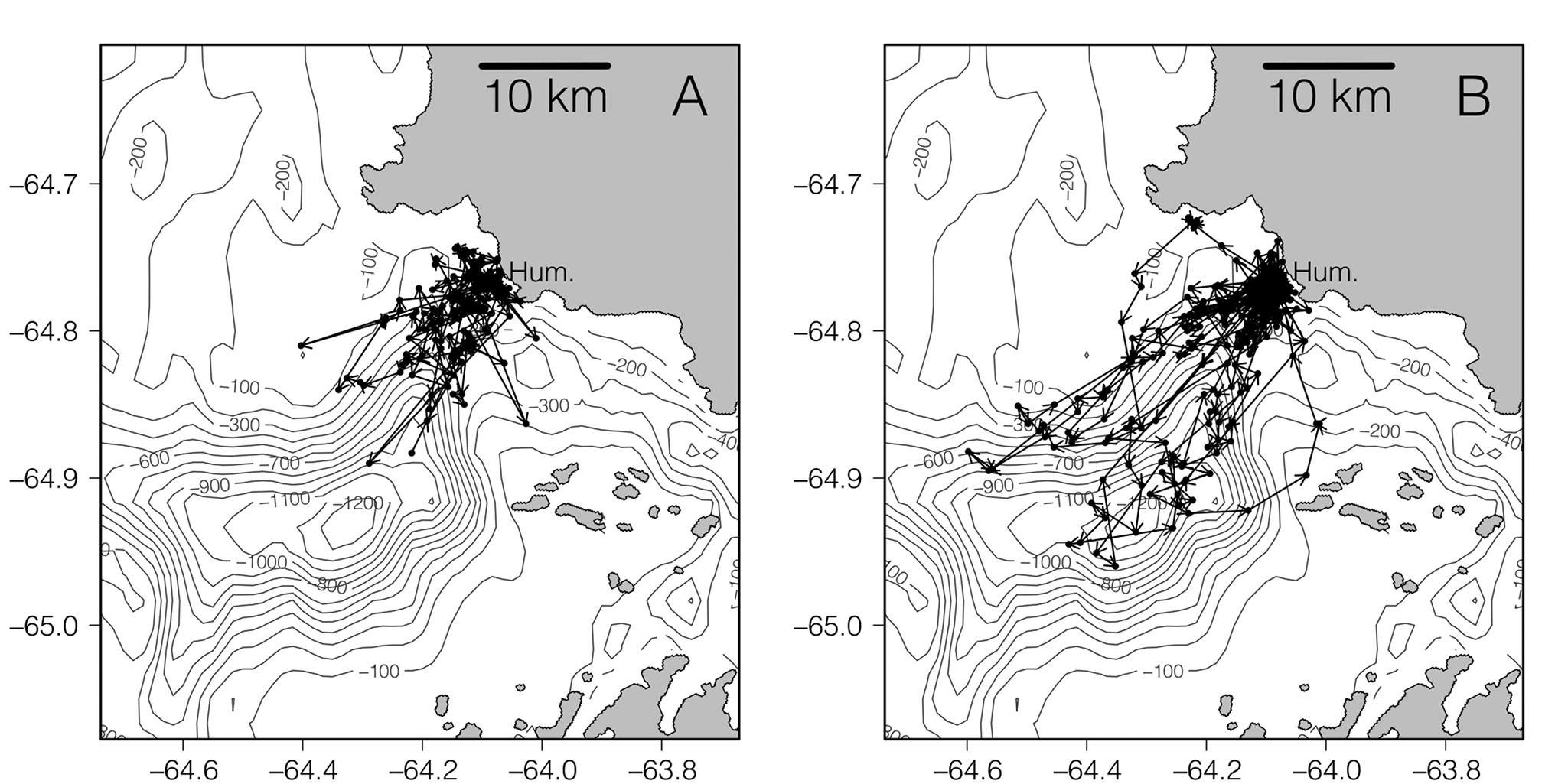

Supplement: Figure S1 — Filtered Adélie penguin satellite tracks from January 2011. Panels A and B are tracks during diurnal and semidiurnal tidal regimes. (TIF) [file pone.0055163.s001.tif]
